# HSV-2 Manipulates Autophagy through Interferon Pathway: A Strategy for Viral Survival

**DOI:** 10.3390/v16091383

**Published:** 2024-08-29

**Authors:** Debashree Dass, Anwesha Banerjee, Kishore Dhotre, Vaishnavi Sonawane, Ashwini More, Anupam Mukherjee

**Affiliations:** Division of Virology, ICMR-National Institute of Translational Virology and AIDS Research, Pune 411026, India; debashree650@gmail.com (D.D.); banerjee.anwesha1991@gmail.com (A.B.); kishoredhotre27@gmail.com (K.D.); rrvbsonawane@gmail.com (V.S.); ashwini05.s@gmail.com (A.M.)

**Keywords:** HSV-2, autophagy, interferon pathway, JAK-STAT pathway, MAPK pathway

## Abstract

Autophagy, an evolutionarily conserved cellular process, influences the regulation of viral infections. While the existing understanding indicates that Herpes Simplex Virus type 2 (HSV-2) maintains a basal level of autophagy to support its viral yield, the precise pathways governing the induction of autophagy during HSV-2 infection remain unknown. Therefore, this study aims to explore the role of type I interferons (IFN-I) in modulating autophagy during HSV-2 infection and to decode the associated signaling pathways. Our findings revealed an interplay wherein IFN-I regulates the autophagic response during HSV-2 infection. Additionally, we investigated the cellular pathways modulated during this complex process. Exploring the intricate network of signaling events involved in autophagy induction during HSV-2 infection holds promising therapeutic implications. Identifying these pathways advances our understanding of host–virus interactions and holds the foundation for developing targeted therapeutic strategies against HSV-2. The insight gained from this study provides a platform for exploring potential therapeutic targets to restrict HSV-2 infections, addressing a crucial need in antiviral research.

## 1. Introduction

Herpes Simplex Virus-2 (HSV-2) stands as one of the most prevalent viruses within the human population, impacting approximately 16% of individuals aged 15–49 globally [1,2]. This double-stranded DNA virus is recognized for its capacity to induce genital herpes. Manifestations include cutaneous and mucosal lesions, ocular complications leading to blindness, and in severe cases, potentially life-threatening encephalitis [3,4,5,6]. HSV-2 exhibits the ability to be vertically transmitted from infected mothers to neonates [7]. Furthermore, individuals infected with HSV-2 face an increased susceptibility to Human Immunodeficiency Virus (HIV) acquisition [8]. The establishment of latency within neurons, coupled with intermittent reactivation from these cellular reservoirs, marks the chronic nature of HSV-2 infections, persisting throughout the lifetime of the host. Recurrent events of symptomatic manifestations coincide with viral shedding, which occurs in both symptomatic and asymptomatic carriers. The latter, specifically asymptomatic shedding, poses a significant concern as it sustains viral transmission, thereby contributing to the persistence of the infection within the population [9]. While antiviral drugs have been established for the treatment of HSV-2, they primarily serve to inhibit viral replication without effectively restricting the spread of the virus or providing a cure [10]. Hence, it is crucial to engage in research approaches directed toward elucidating the complex mechanisms and pathways involved in HSV-2 infection. A detailed understanding of these processes is essential for the development of effective and innovative therapeutic strategies against HSV-2 infections, ultimately addressing the limitations of current treatment modalities.

Autophagy, an evolutionarily conserved cellular process, serves as a pivotal mechanism for the degradation of unwanted cytosolic components, as well as viruses and viral constituents during viral infections [11,12]. The initiation of autophagy is marked by the sequestration of cytoplasmic proteins and damaged organelles, enveloping them within a distinctive cup-shaped double-membraned structure known as the phagophore. This isolation membrane exhibits progressive growth as it encompasses the enclosed contents, ultimately giving rise to autophagosomes [13]. Subsequent fusion of these autophagosomes with lysosomes results in the formation of autolysosomes, wherein the cytosolic components are degraded, thereby maintaining cellular homeostasis [14]. While a basal level of autophagy is constitutively active in all cells, its elevation is observed under conditions of cellular stress, including oxidative stress, hypoxia, starvation, inflammation, and viral infections [15]. Remarkably, autophagy functions as an antiviral mechanism, contributing to cellular survival. Specifically, under conditions of viral infection, autophagy serves to protect the cell by effectively eliminating the invading virus [16,17]. However, the complex relationship between viruses and autophagy reveals an interconnection. Extended periods of interaction have enabled viruses to evolve strategies to manipulate autophagy, thereby enhancing their replication capabilities [18,19]. For instance, the encephalomyocarditis virus (EMCV) strategically utilizes autophagy vesicles as specialized sites for RNA replication [20]. In the case of herpes simplex virus type 2 (HSV-2), autophagy assumes a protective role, as its overexpression has been linked to increased viral infection. Therefore, autophagy emerges as an important factor in HSV-2 replication dynamics, highlighting the complex interaction between autophagy and viral pathogens.

Type I Interferons (IFNs) are cytokines renowned for their diverse antiviral and immunomodulatory activities [21]. Upon activation, they elicit an antiviral state within cells, directing various cellular processes, notably autophagy, to safeguard against viral threats—a phenomenon well-established across diverse cell lines [22]. While the classical induction of the type I interferon pathway involves the activation of the JAK-STAT signaling cascade, emerging evidence highlights the pivotal role of the MAPK pathway in regulating autophagic responses [23,24]. Additionally, several investigations have reported the modulation of the PI3K/AKT/mTOR pathway by Type I Interferons for induction of autophagy [25,26]. Despite this wealth of knowledge, a critical gap remains unaddressed: the correlation between the IFN pathway and the regulation of autophagy specifically during HSV-2 infection has yet to be explored.

In this study, we reveal, for the first time, the complex interplay between HSV-2 and autophagy through the Type I Interferon signaling pathway. Our findings highlight the central involvement of the JAK-STAT pathway in mediating the induction of autophagy during HSV-2 infection. Furthermore, we also investigate the inhibition of MAPK pathway and mTOR upon type I interferon activation within macrophages during HSV-2 infection. This study not only advances our understanding of the complex mechanisms governing host–virus interactions but also sheds light on the previously unexplored interactions between Type I Interferons, autophagy, and HSV-2 pathogenesis.

## 2. Materials and Methods

### 2.1. Cell Culture

The human monocytic/macrophage cell line THP-1 (ATCC: TIB-202™, ATCC, Manassas, VA, USA) was utilized to investigate the kinetics of autophagy induction. Cells were cultured in RPMI-1640 medium (Gibco, Waltham, MA, USA), supplemented with 1% penicillin-streptomycin antibiotics (Gibco, Waltham, MA, USA), 20 mM HEPES (4-(2-hydroxyethyl)-1-piperazineethanesulfonic acid; Gibco, Waltham, MA, USA), 1 mM sodium pyruvate (Gibco, Waltham, MA, USA), and 10% fetal bovine serum (FBS; Invitrogen, Waltham, MA, USA). Differentiation of THP-1 cells into macrophages was achieved by administering 10 nM Phorbol 12-myristate 13-acetate (PMA; Sigma-Aldrich, St. Louis, MO, USA) for 24 h, followed by a 24–48 h resting period in PMA-free complete RPMI media.

Vero cells (African green monkey kidney epithelial cells) (ATCC: CCL-81™; Manassas, VA, USA) were employed for the production and expansion of the HSV-2 virus. Vero cells were cultured in Dulbecco’s modified Eagle’s medium (DMEM; Gibco, Waltham, MA, USA) supplemented with antibiotics and 10% fetal bovine serum.

### 2.2. Virus Propagation

HSV-2 (ATCC: VR-734D™, ATCC, Manassas, VA, USA) propagation was carried out in 2% FBS serum-starved Vero cells for 2–3 days. Virus titers were determined using a plaque assay following established protocols [27].

### 2.3. IFN Studies

To study the kinetics of autophagy induction via the Type I Interferon pathway, THP-1 cells (2 × 10^6^) were mock-treated or treated with 1000 IU/mL concentration of Human IFN-α IFN-α2B (H6166; Sigma-Aldrich, St. Louis, MO, USA), which was dissolved in PBS. THP-1 cells were also treated with 10 ug/mL of IFN inhibitor IFNAR2 Monoclonal Antibody (MMHAR-2: 213851; Invitrogen, Waltham, MA, USA) for IFN studies.

### 2.4. SYBR-Green PCR

Total RNA, isolated via the TRIzol organic extraction method, served as the template for cDNA preparation. The SuperScript™ III First-Strand Synthesis System (18080–051; Invitrogen, Waltham, MA, USA) was employed for reverse transcription of the isolated RNA to generate complementary DNA (cDNA). The SuperScript™ III-derived cDNA from HSV-2-infected THP-1 cells was utilized in real-time PCR to amplify the viral gene UL30. The 2× GoTaq^®^ qPCR SYBR Green Master Mix (A6001; Promega, Madison, WI, USA) and 200 nM primers specific to the target gene were employed in the PCR reaction. Primer sequences for the target genes were designed for this study (Table 1). The PCR reaction master mix included 2× SYBR Green Master Mix, forward and reverse primers, cDNA aliquot, and nuclease-free water, resulting in a total volume of 20 µL for each master mix. Real-time PCR was performed on a PCR machine with appropriate cycling conditions (denaturation, annealing, and extension) specific to the designed primers. The obtained data were subjected to analysis. All qPCR assays were performed according to the MIQE guidelines. GAPDH was utilized as an internal control for normalization of the viral genes. The expression levels of target genes were normalized to the reference gene GAPDH to account for variations in RNA input and reverse transcription efficiency.

### 2.5. ELISA

After designated time points, the supernatants were collected for subsequent analysis. The concentration of Type I interferons (IFN-α and IFN-β) in the cell lysates was determined using Krishgen Biosystems GENLISA™ Human Interferon Alpha and Human IFN-beta GENLISA™ ELISA kit following the manufacturer’s protocol (Cerritos, CA, USA). Briefly, cell lysate samples were added to ELISA plates coated with antibodies specific to Type I interferons. Detection antibodies and substrates were used to quantify the interferon levels, and absorbance was measured at the appropriate wavelength. The ELISA data were analyzed to assess the production of Type I interferons over time, providing insights into the cellular response during HSV-2 infection.

### 2.6. Immunoblotting

Cells were harvested, and cell lysates were prepared using a lysis buffer containing protease inhibitors to preserve protein integrity. Samples, along with a protein ladder, were loaded onto 10% SDS-PAGE gels and subjected to electrophoretic separation. The separated proteins were then transferred onto a polyvinylidene fluoride (PVDF) membrane, which was activated in methanol for 5 min. The PVDF membrane was blocked with 5% non-fat dry milk to prevent non-specific antibody binding. Membranes were incubated with primary antibodies overnight at 4 °C. Rabbit monoclonal JAK1 (1:1000), p-STAT1 (1:1000), STAT1 (1:1000), STAT2 (1:1000), IRF9 (1:1000), LC3I/II (1:500); SQSTM1/p62 (1:1000); BECLIN1 (1:1000), ATG5 (1:1000), ATG7 (1:1000), ATG16L1 (1:1000), LAMP1 (1:1000), p-ERK1/2 (1:1000), ERK1/2 (1:1000), 4E-BP1 (1:1000), p-mTOR (1:1000), mTOR (1:1000) antibodies (Cell Signaling Technology, Danvers, MA, USA) or mouse HSV-2 ICP 8 (1:1000; Abcam, Cambridge, UK) were diluted in blocking buffer. After washing the membrane three times, it was incubated with anti-rabbit or anti-mouse HRP-conjugated secondary antibodies (1:2000; Cell Signaling Technology, Danvers, MA, USA) at room temperature for 1 h. Protein bands were visualized using the HRP substrate Pierce™ ECL Western Blotting Substrate (ThermoFisher Scientific, Waltham, MA, USA) in the ChemiDoc imaging system (BioRad, Hercules, CA, USA). The protein band intensities were quantified using ImageJ software v1.53a (NIH, Bethesda, MD, USA). The obtained values were normalized to the loading standard, GAPDH (HRP-tagged; Santa Cruz Biotechnology, Dallas, TX, USA), to account for variations in protein loading. The normalized protein expression data were analyzed to elucidate the dynamics of key signaling molecules and cellular proteins in response to experimental conditions.

### 2.7. Apoptosis Analysis

#### 2.7.1. Quantification of Bcl-2 and Bax Gene Expression

Cells were harvested, and total RNA was extracted using the TRIzol organic extraction method. The extracted RNA served as the template for cDNA preparation. Reverse transcription of the isolated RNA was performed using the SuperScript™ III First-Strand Synthesis System (18080–051; Invitrogen, Waltham, MA, USA) to generate complementary DNA (cDNA). Quantitative PCR was conducted with the 2× GoTaq^®^ qPCR SYBR Green Master Mix (A6001; Promega, Madison, WI, USA) and specific primers for Bcl-2, Bax, and GAPDH, the latter serving as a housekeeping gene. The relative expression ratios of Bcl-2 to Bax were calculated using the ΔΔCt method to assess apoptosis regulation.

#### 2.7.2. TUNEL Assay for Apoptosis Detection

Apoptosis was further analyzed using the TUNEL assay kit (C10618; Invitrogen, Waltham, MA, USA). THP-1 cells treated with PMA were fixed in 4% paraformaldehyde in PBS for 15 min and permeabilized with 0.1% Triton X-100 in PBS for 5 min. After washing with PBS, the cells were incubated with the TUNEL reaction mixture, containing terminal deoxynucleotidyl transferase (TdT) enzyme and a fluorescent nucleotide, for 60 min at 37 °C. Camptothecin was used as a positive control. Following incubation, the cells were washed and counterstained with DAPI. Apoptotic cells were visualized and quantified using a Leica SP8 Spectral Confocal microscope.

#### 2.7.3. Flow Cytometric Analysis of Apoptotic Cell Populations

Apoptotic cell populations were additionally analyzed using the Dead Cell Apoptosis Kit (V13242; Invitrogen, Waltham, MA, USA). Cells were harvested, washed with PBS, and resuspended in 100 µL of Annexin V binding buffer. The cells were then stained by incubating with 5 µL of Annexin V-FITC and 5 µL of Propidium Iodide (PI) for 15 min at room temperature in the dark. After incubation, the cells were diluted with 400 µL of Annexin V binding buffer and analyzed using a BD FACSAria™ Fusion Cell Sorter (Becton-Dickinson, Franklin Lakes, NJ, USA). A total of 10,000 events were acquired to determine fluorescence intensity, and data analysis was performed using Cell Quest software. The flow cytometric analysis distinguished live cells (Annexin V-negative, PI-negative), early apoptotic cells (Annexin V-positive, PI-negative), and late apoptotic or necrotic cells (Annexin V-positive, PI-positive).

### 2.8. Statistical Analysis 

Mean and standard deviations of a minimum of three independent experiments were represented as the final results of the study in the manuscript. One-way ANOVA followed by post hoc tests was performed to evaluate the differences between the means. The *p*-value is represented in asterisks and was observed to be <0.05 in all the experiments conducted for this study. The number of asterisks is indicative of the significance level, i.e., * *p* < 0.05, ** *p* < 0.01, *** *p* < 0.001. The post hoc tests for multiple comparisons following the Bonferroni and Newman–Keuls methods involving the mock-infected and the infected experimental groups were conducted using GraphPad Prism version 5.01 (https://www.graphpad.com, accessed on 9 June 2024). 

## 3. Results

The integral role of autophagy in sustaining HSV-2 activity is well-established, with the suppression of autophagy during HSV-2 infection resulting in a diminished virus yield, as corroborated by numerous studies [28,29]. However, the precise pathway through which HSV-2 induces and maintains autophagy for its benefit remains unexplored. As numerous studies have established the role of type I interferons on autophagy regulation across various cell lines [22,30], we investigated whether the type I interferon pathway is activated to induce autophagy during HSV-2 infection.

### 3.1. HSV-2 Activates Type I Interferon Pathway in Macrophages

**Figure 1:**
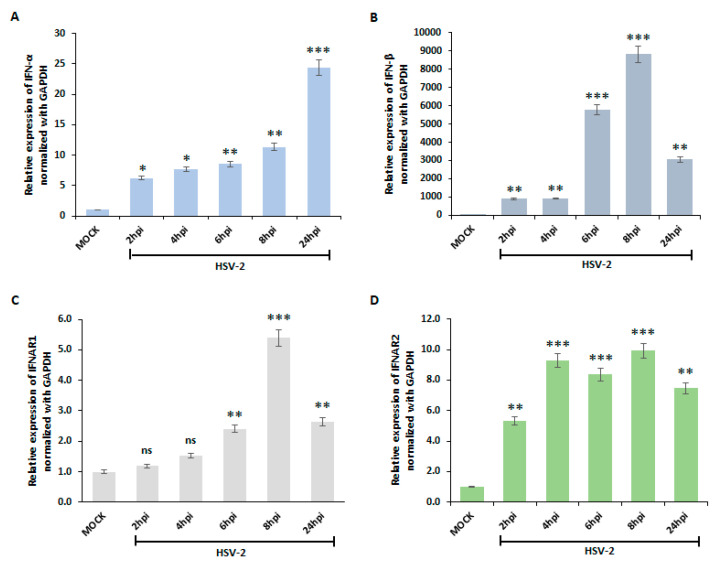
Activation of Interferon Pathway during HSV-2 Infection. Relative expression levels of type I interferon (**A**) IFN-α and (**B**) IFN-β, and their receptors (**C**) IFNAR1 and (**D**) IFNAR2 mRNA in macrophages during HSV-2 infection (MOI 1) compared to mock-infected controls at various time points (2, 4, 6, 8, and 24 hpi), as determined by quantitative Reverse Transcription-Polymerase Chain Reaction (qRT-PCR) analysis. Gene expression levels were normalized to GAPDH. Results are presented as the means and standard deviations from three independent experiments. ns, nonsignificant; *, *p* < 0.05; **, *p* < 0.01; ***, *p* < 0.001.

To determine whether the type I interferon pathway is activated upon HSV-2 infection, THP-1 monocyte cells were differentiated to macrophages with PMA then infected with HSV-2 (MOI 1) and collected at different time points 2 hpi, 4 hpi, 6 hpi, 8 hpi, and 24 hpi (hours post-infection) to quantify the gene expression of IFN-α (Alpha) and IFN-β (Beta) and their receptors (IFNAR1 and IFNAR2). We observed the activation of type I interferons increases significantly over time following HSV-2 infection (Figure 1).

The mRNA expression of IFN-α increases with the progression of HSV-2 infection, showing the highest fold change at the 24 hpi time point (Figure 1A), whereas IFN-β exhibited an earlier and more robust upregulation, with statistically significant elevation as early as 6 h post-infection, reaching a maximum 8 × 10^4^ fold change at 8 hpi and reduction to 3 × 10^4^ fold change at 24 hpi indicating release of IFN-β (Figure 1B). The mRNA expression of IFNAR1 (Interferon Alpha/Beta Receptor 1) and IFNAR2 (Interferon Alpha/Beta Receptor 2) increases gradually with the progression of HSV-2 infection (Figure 1C,D). Collectively, the mRNA expression of type I interferons and their receptors revealed a statistically significant increase following the HSV-2 infection compared to the mock-infected group. The expression level of the receptors IFNAR1 and IFNAR2 were upregulated by 5–10 fold when infected with HSV-2 at 8 hpi compared to the mock-infected group and then downregulated by 2–7 fold at 24 hpi respectively indicating the release of the IFN receptors. HSV-2 UL30 expression was examined to determine the viral infection (Figure S1). 

**Figure 2:**
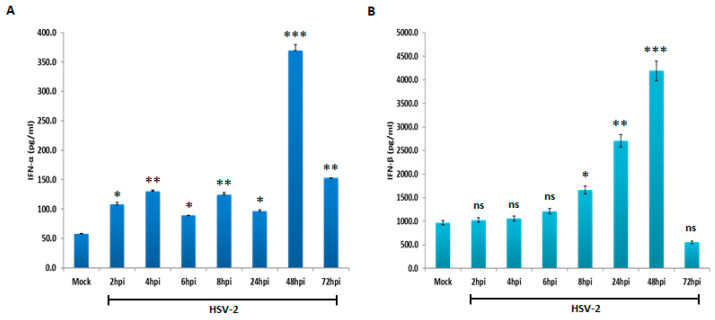
Release of Type I Interferons During HSV-2 Infection. Enzyme-Linked Immunosorbent Assay (ELISA) depicting the dynamic expression of (**A**) IFN-α and (**B**) IFN-β in macrophages at various time points (2, 4, 6, 8, 24, 48, and 72 hpi) during HSV-2 infection (MOI 1) compared to the mock-infected group. The error bar signifies the means and standard deviations from three independent experiments. The data represented have been statistically analyzed. Note: ns, nonsignificant; *, *p* < 0.05; **, *p* < 0.01; ***, *p* < 0.001.

To validate the results of gene expression analysis, ELISA was performed to check the expression of type I interferons after infection with HSV-2. Remarkably, the secreted levels of both interferons (IFN-α and IFN-β) exhibit a gradual increase over the time following HSV-2 infection, with a statistically significant surge observed from 8 h post-infection onwards, with the highest levels of expression observed at the later time points from 24 hpi to 48 hpi and downregulation in their expression after 48 hpi (Figure 2A,B). While both IFN-α and IFN-β displayed similar expression patterns, the release of IFN-β was significantly higher in HSV-2-infected medium compared to IFN-α. Our result revealed the levels of IFN-α and IFN-β mRNA increased over time after HSV-2 infection, and their protein expression reached the highest levels at the later time points of 24 hpi, and 48 hpi, and reduced at 72 hpi.

These results suggest that HSV-2 infection triggers the activation of the type I interferon response pathway, leading to the increased production of IFN-α and IFN-β, over time. This induction of interferons and their receptors likely plays a role in the induction of autophagy upon HSV-2 infection.

### 3.2. Type I IFN Pathway Mediates Downstream Signaling through the JAK-STAT Pathway upon HSV-2 Infection

During viral infection, the type I interferon pathway is activated, resulting in the release of IFN-α and IFN-β. These cytokines, in turn, bind to the IFN receptor (IFNAR1 and IFNAR2), initiating a cascade of events involving JAK1 and TYK2 activation. The activated JAK1 catalyzes the phosphorylation of STAT1 and STAT2. The phosphorylated STATs form a heterotrimeric complex with the IRF9. This complex, termed the IFN-stimulated gene factor 3 (ISGF3), translocates to the nucleus and binds to interferon-stimulated response elements (ISREs) in the promoters of interferon-stimulated genes (ISGs), thereby inducing an antiviral state by activating processes that impede viral propagation, such as autophagy [31].

**Figure 3:**
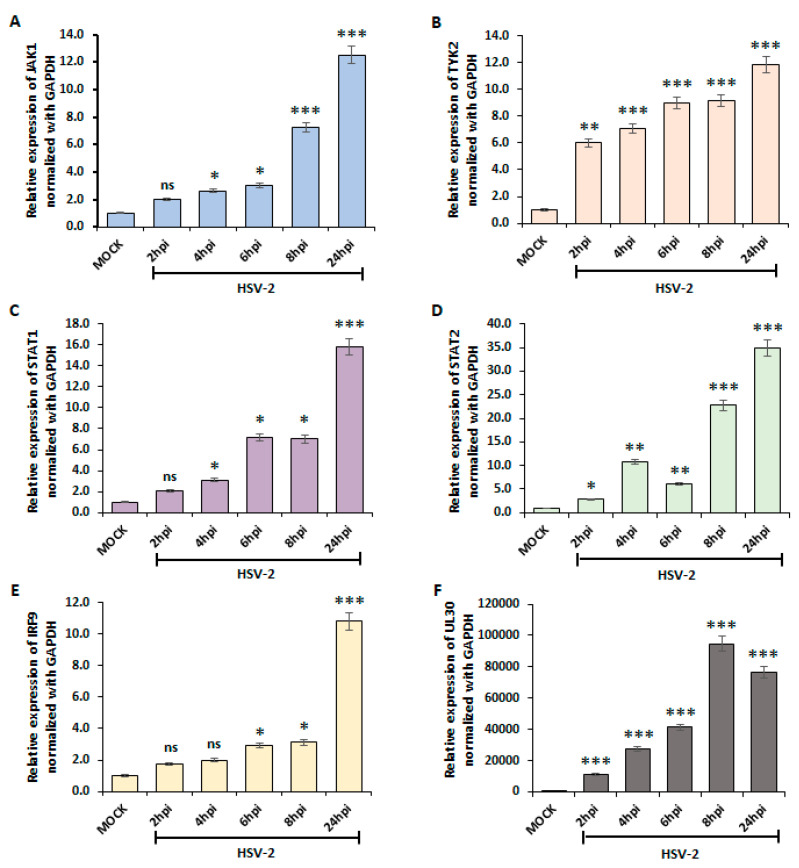
Expression of JAK-STAT pathway during HSV-2 infection. THP-1 cells were infected with 1 MOI of HSV-2 at different time points (2, 4, 6, 8, and 24 hpi). The mRNA expressions of (**A**) JAK1, (**B**) TYK2, (**C**) STAT1, (**D**) STAT2, and (**E**) IRF9 were assessed at different time points of HSV-2 infection and were compared to mock. (**F**) HSV-2 UL30 expression was examined to determine the viral infection. Results are presented as the means and standard deviations from three independent experiments. ns, nonsignificant; *, *p* < 0.05; **, *p* < 0.01; ***, *p* < 0.001.

To investigate whether the type I interferon pathway activates the JAK-STAT pathway for downstream signaling during HSV-2 infection, we conducted qPCR analysis to evaluate the gene expression levels of key components in the JAK-STAT pathway, including JAK1, TYK2, STAT1, STAT2, and IRF9 (Figure 3). The gene expression of JAK1, TYK2, STAT1, STAT2, and IRF9 exhibited a time-dependent increase in their mRNA levels following HSV-2 infection. Notably, a significant induction was observed, commencing with an approximately 2-fold increase at 2 hpi, and progressively escalating with increasing time points of HSV-2 infection relative to the mock-infected controls (Figure 3A–F). This observation indicates a moderate initial induction of the JAK-STAT pathway components in response to early HSV-2 infection. The highest fold change was observed at 24 hpi where all genes JAK1, TYK2, STAT1, STAT2, and IRF9 showed upregulation of 10-fold or greater. The elevated expression of these signaling molecules suggests their involvement in mediating the downstream effects of type I interferon signaling.

**Figure 4:**
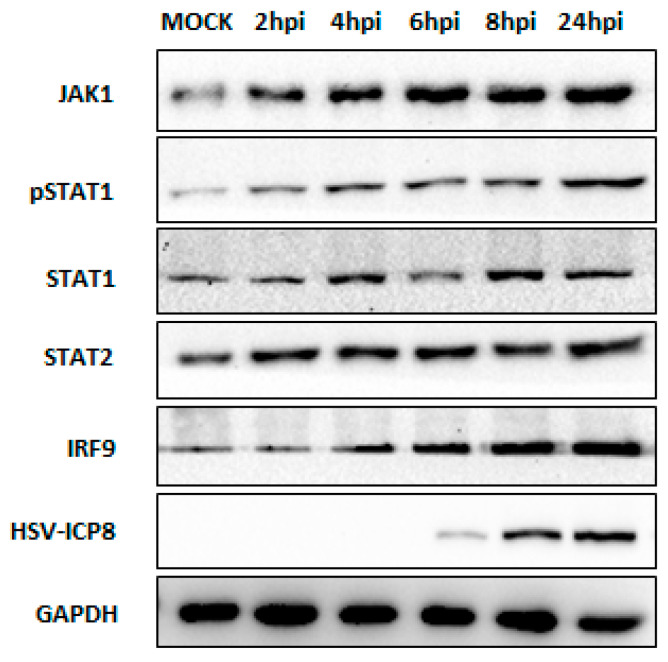
HSV-2 infection activates the JAK-STAT signaling cascade in macrophages. Expressions of JAK1, p-STAT1, STAT1, STAT2, and IRF9 were examined upon PMA differentiated monocyte-derived THP-1 macrophages infected with HSV-2 (1 MOI) at different time points (2–24 hpi). Virus infection was confirmed by analyzing HSV-2 ICP8 expression in the same cell lysates. GAPDH was used as an internal control. Results shown are representative of at least three independent experimental replicates.

To validate the results of qPCR, we performed Western blot analysis to evaluate the protein expression levels of key JAK-STAT pathway components in THP-1 cells infected with HSV-2 at different time points, in comparison with the mock-infected group (Figure 4). The analysis revealed increased protein expression of JAK1, phosphorylated STAT1 (p-STAT1), total STAT1, and STAT2 over time following HSV-2 infection, with the highest expression observed at 24 hpi (Figure 4). As the mRNA level of the genes of the JAK-STAT pathway rises from the initial time points of infection, it is possible that the translation process starts earlier, and the peak level of protein expression observed at 24 hpi could be a cumulative result of the translational process that began earlier, after the initial mRNA induction. Similarly, IRF9, a component of the ISGF3 complex, also showed an increasing trend in its protein level over time after HSV-2 infection, further supporting the activation of the JAK-STAT pathway in response to HSV-2 infection, and the potential formation of the ISGF3 complex (Figure 4). Despite the induction of the antiviral response, the levels of the HSV-2 ICP-8 protein, a key viral protein involved in viral replication, exhibited a significant increase at later time points (Figure 4). This observation suggests that HSV-2 employs mechanisms to counteract the host antiviral defenses and facilitate its replication process.

These results demonstrate that HSV-2 infection triggers the production of type I interferons, which in turn activates the JAK-STAT pathway, a crucial signaling cascade downstream of the type I interferon response. The upregulation of key components, including JAK1, TYK2, STAT1, STAT2, and IRF9, at both the mRNA and protein levels is observed over the course of the HSV-2 viral infection. This activation likely leads to an antiviral state within the cells; however, HSV-2 appears to manipulate these host defenses, as evidenced by the increased levels of the viral ICP-8 protein, highlighting the interplay between the virus and the host antiviral mechanisms.

### 3.3. Type I IFN Induces Autophagy upon HSV-2 Infection

**Figure 5:**
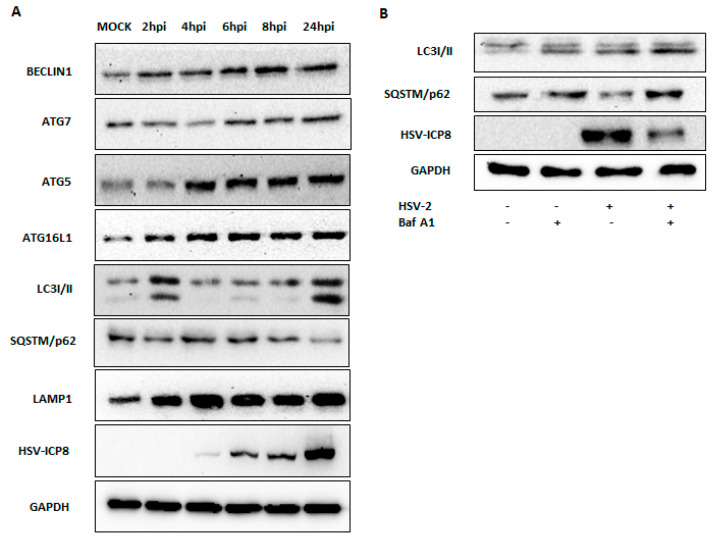
Expression of autophagy markers during HSV-2 infection: (**A**) Western blot analysis indicates the protein expression level of key autophagy markers at different time points of HSV-2 infection (MOI 1) compared with mock-infected cells. BECLIN1, ATG7, ATG5, ATG16L1, LC3-I/II, and LAMP1 protein expression increased along with persistent viral infection, while p62 expression gradually decreased at later time points. HSV-2 infection was confirmed by assessing ICP8 expression in the same cell lysates. GAPDH was used as an internal control. (**B**) The expression of LC3-I/II and p62 was analyzed to assess autophagy activation in monocyte-derived macrophages treated with Bafilomycin A1 (Baf A1) and/or infected with HSV-2, compared to mock-infected cells. HSV-2 infection was confirmed by assessing the expression of the HSV-2 ICP8 protein in the same cell lysates, and GAPDH was used as an internal control. Results shown are representative of at least three independent experimental replicates.

To determine whether the HSV-2 infection triggers the activation of autophagy, PMA differentiated THP-1 cells were infected with HSV-2 and collected at different time points (2–24 hpi) and compared to the mock-infected group. Immunoblot analysis using specific antibodies revealed a time-dependent increase in the expression of key autophagy markers following HSV-2 infection when compared to mock-infected cells (Figure 5). Importantly, the protein levels of BECLIN1, ATG7, ATG5, ATG16L1, LC3-I/II, SQSTM (p62), and LAMP1 exhibited a gradual upregulation, peaking at the 24 hpi time point (Figure 5). 

Previous studies have shown that autophagy activation can be determined by analyzing LC3 and p62 levels, with an increase in LC3-II and a decrease in p62 serving as indicators of autophagy activation [32]. To validate autophagy induction following HSV-2 infection, we compared LC3-I/II and p62 protein levels in HSV-2-infected cells with those in mock-treated cells, a known autophagy inhibitor bafilomycin A1-treated cells, and HSV-2-infected cells treated with bafilomycin A1. Our results demonstrated that LC3-II levels were elevated in HSV-2-infected cells compared to mock-treated cells. Conversely, p62 levels were reduced in HSV-2-infected cells compared to mock-treated cells, bafilomycin-treated cells, and HSV-2-infected cells treated with bafilomycin, indicating that autophagy was activated upon HSV-2 infection. The increased LC3-II and p62 levels observed in HSV-2-infected cells treated with bafilomycin suggest that bafilomycin inhibits autophagy by preventing the fusion of autophagosomes with lysosomes and blocking the degradation of p62 (Figure 5B).

**Figure 6:**
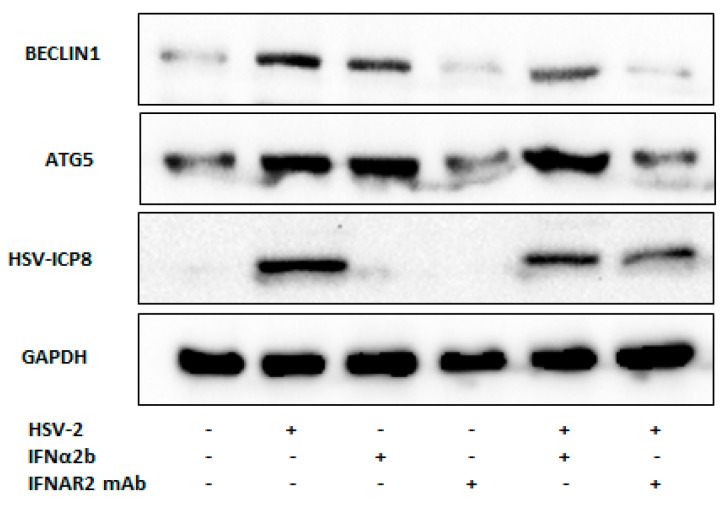
Type I Interferon activates autophagy during HSV-2 infection. Cells were pre-treated with IFN-α2B or a neutralizing IFNAR2 mAb as an inhibitor of type I IFNs, followed by mock- or HSV-2 infection (MOI 1). The expression of BECLIN1 and ATG5 was examined to assess the induction of autophagy in IFN-α2B-treated and/or HSV-2-infected monocyte-derived macrophages, and in cells treated with the IFN inhibitor alone or with HSV-2 infection, compared to mock infection. The expression of HSV-2 ICP8 was assessed to confirm viral infection in the same cell lysates. GADPH was used as an internal control. Results shown are representative of at least three independent experimental replicates.

It is well established that type I interferon is responsible for the activation of autophagy in many cell types [22]. Therefore, to elucidate whether type I interferon is involved in the induction of autophagy upon HSV-2 infection, we checked the protein expression level of key autophagy components, BECLIN1 and ATG5 in IFN-α2B alone or in combination with HSV-2, compared to mock- or only virus-infected conditions at 24 hpi (Figure 6). The protein expression levels of these autophagy markers in IFN-α2B-treated cells were found to be similar to those observed in HSV-2-infected cells (Figure 6). Notably, the protein expression levels were further augmented in cells co-treated with IFN-α2B and HSV-2, compared to mock-infected, HSV-2-infected, or IFN-α2B-treated cells alone. The protein expression of these autophagy markers, including BECLIN1, involved in autophagy initiation, and ATG5, involved in autophagosome formation, was inhibited upon treatment with an IFN inhibitor (IFNAR2 mAb) compared to HSV-2-infected cells (Figure 6). IFNAR2 mAb neutralizes the human IFN-α receptor by interacting with its extracellular domain, binding with high affinity and blocking the biological action of Type I IFNs. Inhibition of these autophagy markers was significantly more pronounced when HSV-2-infected cells were treated with IFN inhibitor, compared to HSV-2-infected or IFN inhibitor alone, highlighting the probable crosstalk between type I interferon and autophagy during HSV-2 infection. These proteins are crucial components of the autophagy machinery, suggesting that HSV-2 infection triggers the induction of the autophagic pathway via type I interferon signaling. Despite the activation of type I interferon-induced autophagy, the expression level of the viral protein ICP-8 increased over time, which indicates that the HSV-2 can modulate the type I interferon-induced autophagy for its survival.

### 3.4. Type I IFN Induces Autophagy through Inhibition of ERK1/2 and mTOR

**Figure 7:**
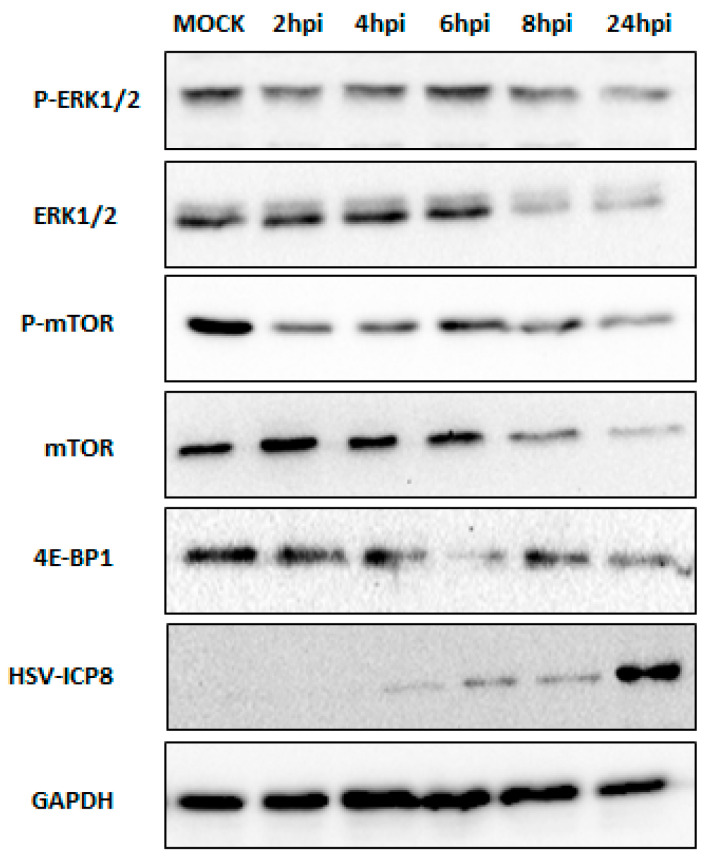
Expression of negative regulators of autophagy during HSV-2 infection. Western blot analysis of proteins associated with the MAPK and PI3K/AKT/mTOR pathways indicates significant downregulation at different time points of HSV-2 infection (MOI 1) compared with mock-infected cells. The expression of p-ERK1/2 (p-p44/42 MAPK), ERK1/2 (p44/42 MAPK), phospho-mTOR, mTOR, and 4E-BP1 proteins was suppressed as the viral infection progressed, with maximum inhibition observed at 24 hpi. HSV-2 infection was confirmed by assessing ICP8 expression in the same cell lysates. GAPDH was used as an internal control. Results shown are representative of at least three independent experimental replicates.

The MAPK/ERK1/2 signaling pathway is known to restrict autophagy by activating mTOR [33]. However, the regulatory mechanisms governing the type I interferon-induced autophagic response during HSV-2 infection had not been fully explored. Therefore, we assessed the levels of p-ERK1/2 (p-p44/42 MAPK), ERK1/2 (p44/42 MAPK), p-mTOR, mTOR, and the mTOR downstream regulator 4E-BP1 at different time points (2–24 hpi) of HSV-2 infection (Figure 7). 

The expression levels of phosphorylated ERK1/2 (p-ERK1/2) and total ERK1/2 were significantly reduced in cells infected with HSV-2 at 24 h post-infection (hpi) compared to mock-infected cells. This suggests that HSV-2 infection leads to a marked decrease in both the total ERK1/2 protein and its phosphorylated form. The reduction in p-ERK1/2 mirrors the decrease in total ERK1/2, indicating that HSV-2 infection impairs both the expression and activation (phosphorylation) of ERK1/2 as the infection progresses from 2 to 24 hpi. These findings suggest that HSV-2 infection inhibits the ERK1/2 signaling cascade, which could potentially contribute to the activation of autophagy through the inhibition of mTOR, an important regulator of autophagy. Furthermore, monitoring mTOR activity through the protein expression of phosphorylated mTOR (p-mTOR), mTOR, and its downstream target protein EIF4E-BP1 upon HSV-2 infection revealed a decrease in their expression over time, indicative of mTOR inhibition (Figure 7).

**Figure 8:**
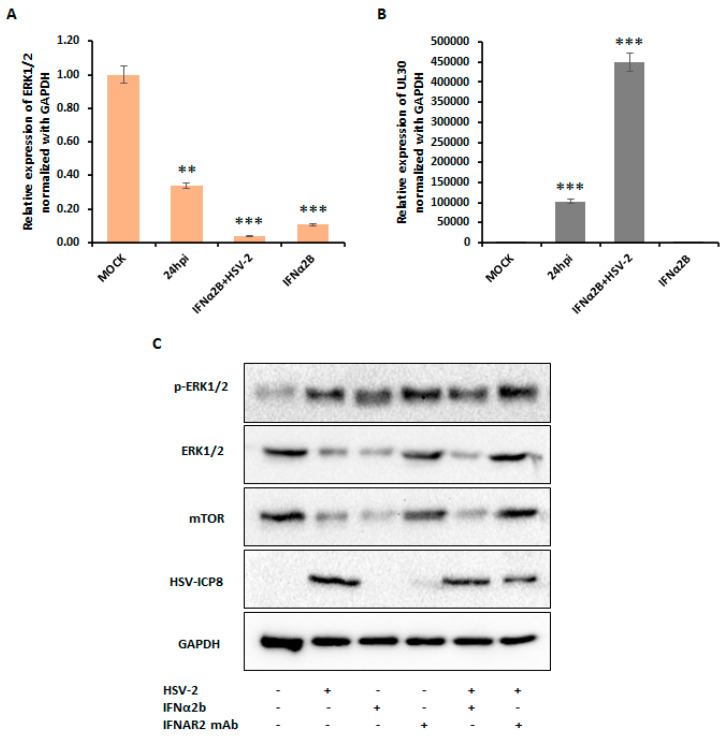
HSV-2-induced type I IFN inhibits ERK1/2 and mTOR expressions. Thp-1 cells were infected with HSV-2 (MOI) and analyzed at 24 hpi. Cells were treated with IFN-α2B alone or in combination with HSV-2 infection. The mRNA expression levels of (**A**) ERK1/2 and (**B**) HSV-2 UL-30 were assessed and compared to the mock-infected group. Error bars represent the means and standard deviations from three independent experiments. **, *p* < 0.01; ***, *p* < 0.001. (**C**) Protein expression levels of p-ERK1/2, ERK1/2, and mTOR were assessed in cells pre-treated with IFN-α2B or a neutralizing IFNAR2 mAb as an inhibitor of type I IFNs, followed by mock or HSV-2 infection. The expression of HSV-2 ICP8 was assessed to confirm viral infection in the same cell lysates. GAPDH was used as an internal control. Results shown are representative of at least three independent experimental replicates.

To elucidate the mechanism by which type I IFN induces autophagy, we investigated the roles of ERK1/2 and mTOR upon HSV-2 infection. THP-1 cells were infected with HSV-2 and examined at 24 hpi, a time point when type I IFN expression is maximal, leading to an increase in autophagy. These cells were compared to cells treated with IFN-α2B alone and cells co-treated with IFN-α2B and HSV-2. Our data revealed that HSV-2-infected cells exhibited a 66.3% inhibition of ERK1/2 mRNA expression, whereas overexpression of type I interferon downregulated ERK1/2 mRNA expression by 89.4%. Furthermore, cells co-treated with IFN-α2B and HSV-2 showed a 96.4% inhibition of ERK1/2 mRNA expression, indicating that HSV-2 infection downregulates ERK1/2 mRNA expression through type I interferon signaling (Figure 8A). Effective viral infection was confirmed by examining HSV-2 UL30 expression from the same samples (Figure 8B).

To determine whether type I interferon is involved in the inhibition of mTOR during HSV-2 infection, we examined the protein expression levels of mTOR, p-ERK1, and ERK1/2 in THP-1 cells under various conditions at 24 hpi. These conditions included treatment with IFN-α2B alone, co-treatment with IFN-α2B and HSV-2, and treatment with a neutralizing IFNAR2 mAb (as an IFN inhibitor) with and without HSV-2 infection. These were compared to HSV-2-infected cells and the mock-infected group (Figure 8C). Cells treated with IFN-α2B alone showed a decrease in the protein expression of p-ERK1/2, ERK1/2, and mTOR, similar to the decrease observed in HSV-2-infected cells. This supports the inhibitory effects of type I interferon on these pathways. In contrast, cells treated with the type I interferon inhibitor, with or without HSV-2 infection, showed a recovery in the protein expression of p-ERK1/2, ERK1/2, and mTOR. Notably, HSV-2 infection in cells pre-treated with the IFN inhibitor did not suppress the expression of p-ERK1/2, ERK1/2, and mTOR, unlike HSV-2 infection alone. These results suggest that type I interferon modulates autophagy by inhibiting ERK1/2 activation through the suppression of p-ERK1/2, which in turn inhibits mTOR. Overall, this inhibition may lead to the activation of autophagy during HSV-2 infection.

## 4. Discussion

**Figure 9:**
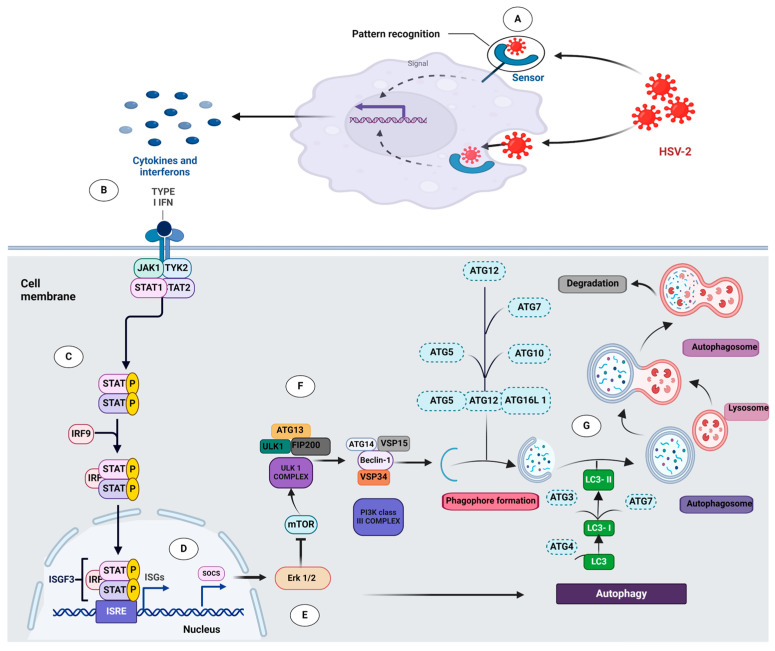
Schematic representation of the key signaling pathways involved in the initiation of autophagy upon HSV-2 infection: (**A**) Upon entry into the host cell, HSV-2 is recognized by pattern recognition receptors, which detect pathogen-associated molecular patterns, leading to the activation of inflammatory pathways and the production of cytokines and interferons [35]. (**B**) Type I interferons (IFN-α/β) bind to their respective receptors (IFNAR1/2), triggering the activation of the Janus kinase/signal transducer and activator of transcription (JAK/STAT) pathway [36]. (**C**) The JAKs (JAK1 and TYK2) phosphorylate STAT1 and STAT2, which subsequently form a heterotrimeric complex with IRF9, known as the IFN-stimulated gene factor 3 (ISGF3). This complex translocates to the nucleus and binds to IFN-stimulated response elements (ISREs) in the promoter regions of IFN-stimulated genes (ISGs), inducing their transcription [31]. (**D**) Among the ISGs, the suppressor of cytokine signaling (SOCS) proteins are produced, which interact with and inhibit components of the Ras–Raf–MEK–ERK cascade, leading to the inhibition of ERK1/2 [37]. (**E**) The inhibition of ERK1/2 results in the downregulation of the mechanistic target of rapamycin complex 1 (mTORC1), a critical regulator of cellular processes, including autophagy [38]. (**F**) The inhibition of mTORC1 triggers the initiation of autophagy through the activation of the ULK1 complex, comprising ULK1, ATG13, FIP200, and ATG101 [39]. (**G**) The autophagy-related genes (ATGs) are activated, leading to the formation of the phagophore and the eventual maturation of the autophagosome, which ultimately fuses with the lysosome to complete the autophagic process [40].

Autophagy, a highly conserved catabolic process, exhibits a dichotomous role during viral infections, functioning both as an antiviral defense mechanism and a viral survival strategy [34]. In the context of HSV-2 infection, studies have indicated a paradoxical relationship, wherein the virus manipulates autophagy to its advantage, highlighting the crucial role autophagy plays in the survival of the virus [28]. Our study aims to elucidate the interrelated pathways regulating autophagy during HSV-2 infection, with a particular focus on the involvement of the type I interferon signaling pathway (Figure 9).

Numerous studies have highlighted the role of the type I interferon pathway in inducing autophagy across diverse cell lines, a key mechanism in the antiviral immune response [41]. However, the involvement of this pathway in autophagy induction during HSV-2 infection has remained unexplored. Our findings demonstrate that type I interferons induce autophagy in macrophages upon HSV-2 infection. Interferons, secreted by immune cells such as macrophages, are activated in response to external stimuli like viral entry [42]. Our data demonstrate that upon HSV-2 infection, activation of type I interferon occurs. An increase in the mRNA level of IFN-α and IFN-β indicates the induction of type I interferons, in response to HSV-2 infection (Figure 1). In addition, type I interferons share a common surface receptor of two IFN-α receptor subunits (IFNAR1 and IFNAR2) [43]. The mRNA expression of these receptors is also elevated upon HSV-2 infection and the induced protein expression of IFN-α and IFN-β upon HSV-2 infection confirms the activation of type I interferons upon HSV-2 infection (Figure 1 and Figure 2). 

Type I interferons are known to induce the Janus kinase-signal transducer and activator of transcription (JAK-STAT) pathway upon binding to their receptors [44]. Our data revealed that HSV-2 infection leads to the activation of the JAK-STAT pathway, as evidenced by the elevated mRNA and protein expression levels of its components, suggesting a link between type I interferon activation and JAK-STAT pathway induction during HSV-2 infection (Figure 3 and Figure 4). The activated JAK-STAT pathway, in turn, promotes the formation of the interferon-stimulated gene factor 3 (ISGF3) complex, which translocates to the nucleus and binds to interferon-stimulated response elements (ISREs) in the promoter regions of interferon-stimulated genes (ISGs) [45]. 

JAK-STAT1 activation promotes IFN-α-induced autophagy, STAT1, and NFκB are required for IFN-α-induced BECLIN1 expression [30]. Another study showed treatment of STAT2-defective mutant Daudi cells did not increase levels of MAP1LC3-II and saw a decrease in SQSTM1, suggesting that IFN-induced autophagy is STAT2-dependent [20]. Our findings further indicate that type I interferon signaling is a potent inducer of autophagy, as evidenced by a significant upregulation of autophagic markers (BECLIN1, ATG7, ATG5, ATG16L1, LC3-I/II, and LAMP1) upon type I interferon treatment. In contrast, treatment with the autophagy inhibitor Bafilomycin used for studying autophagy by inhibiting lysosomal acidification, thereby disrupting the autophagic process and affecting the expression and accumulation of various autophagy-related markers (LC3-II and p62), resulted in an increase in p62 expression and LC3-II, whereas a decrease in p62 levels and increase in LC3-I/II expression level indicates the activation of autophagy following HSV-2 infection (Figure 5 and Figure 6). As initiation of autophagy starts with the formation of PI3K complex, where BECLIN1 is a part of the class III phosphatidylinositol 3-kinase (PI3K) association, this complex is responsible for generating phosphatidylinositol 3-phosphate (PI3P) at the site of autophagosome formation, initiating the nucleation of the autophagosomal membrane [46]. ATG7 activates ATG12, facilitating its conjugation with ATG5 to form the ATG5-ATG12 complex [47]. For elongation and maturation, this complex is associated with ATG16L1. LAMP1 then aids in the fusion of the autophagosome with the lysosome, completing autophagy [48]. LC3-II is a marker for the formation of autophagosomes, while p62 serves as an indicator of the efficiency of autophagic degradation [49]. Bafilomycin A1 is a potent and specific inhibitor of the vacuolar-type H+-ATPase (V-ATPase), an enzyme complex responsible for acidifying intracellular compartments such as lysosomes and endosomes. By inhibiting V-ATPase, bafilomycin disrupts the acidification process within these organelles, which is crucial for their function in processes such as protein degradation and autophagy [50]. The induction of autophagy was observed not only upon IFN-α2B treatment alone but also during HSV-2 infection, with co-treatment of type I interferon and HSV-2 infection leading to further enhancement of autophagy induction. Conversely, treatment with a type I interferon inhibitor, either alone or in combination with HSV-2 infection, reduced autophagy induction, confirming the important role of type I interferon in autophagy regulation during HSV-2 infection (Figure 6). It is already known that HSV-2 causes apoptotic infection in monocytoid cells [51]. We investigated the induction of apoptosis following HSV-2 infection, noting that LC3-I/II expression initially increased during early infection, followed by a decrease, and then another rise at 24 h post-infection (hpi). To determine whether cells were undergoing early apoptosis in conjunction with autophagy, we conducted multiple assays to examine apoptosis induction upon infection (Figure S2). The increased Bcl-2/Bax ratio indicates that HSV-2 infection actively triggers apoptotic pathways in infected cells. The TUNEL assay, which detects DNA fragmentation, a hallmark of apoptosis, showed a significant increase in TUNEL-positive cells in HSV-2-infected samples compared to mock-infected controls. This finding supports our qPCR data, confirming that HSV-2 infection leads to apoptosis in the infected cells. Flow cytometry analysis using Propidium Iodide (PI) and Annexin V staining further confirmed that HSV-2 infection induces cell death. Most cells in the HSV-2-infected group were positive for PI but not for Annexin V, suggesting that pyroptosis, a form of inflammatory cell death, is predominant. The presence of PI-positive/Annexin V-negative cells indicates that these cells are undergoing late apoptosis or necrosis, particularly pyroptosis, rather than early apoptosis, which is typically marked by Annexin V positivity. Overall, these findings clearly demonstrate that HSV-2 infection triggers apoptosis in host cells, as evidenced by the altered Bcl-2/Bax ratio, increased DNA fragmentation, and the cell death patterns observed in flow cytometry. The predominance of PI staining in the FACS analysis suggests that pyroptosis may be a significant form of cell death in HSV-2-infected cells. This indicates that the virus may induce a mixed type of cell death, with pyroptosis being particularly prominent. These findings provide important insights into the pathogenic mechanisms of HSV-2 and its impact on host cell survival.

Type I interferon activates the JAK-STAT pathway and is known to induce ISGs by the ISGF3 complex, one of which is SOCS (Suppressor of Cytokine Signaling), which interacts with and inhibits components of the Ras–Raf–MEK–ERK cascade, which is the upstream activator of ERK1/2 [37,52]. SOCS1 and SOCS7 have been shown to bind to the Ras-Grb2 complex, preventing the activation of Ras and subsequently inhibiting the downstream activation of the ERK1/2 pathway [52]. Interestingly, our data revealed that type I interferon activation leads to the inhibition of the extracellular signal-regulated kinase 1/2 (ERK1/2) pathway, a known upstream activator of the mechanistic target of rapamycin complex 1 (mTORC1) through the suppression of phosphorylation of ERK1/2. Our data demonstrated that IFN-α2B treatment and HSV-2 infection at later time points reduced the expression of p-ERK1/2 and ERK1/2, whereas the expression of ERK1/2 was further inhibited with the co-treatment of HSV-2 and IFN-α2B (Figure 8). In contrast, treatment with type I interferon inhibitor showed the opposite effect on the expression of ERK1/2, displaying the role of type I interferon in the inhibition of ERK1/2 upon HSV-2 infection (Figure 8).

ERK1/2 can control mTOR signaling as it acts upstream of it [22]. Inhibition of the ERK1/2 pathway leads to the downregulation of the mTORC1 complex since ERK1/2 positively regulates mTORC1 activity [53]. Additionally, ERK1/2 modulates other signaling pathways that regulate mTORC1 activity. It can phosphorylate and inhibit TSC2, a negative regulator of mTORC1, by inhibiting the small GTPase RHEB, a direct activator of mTORC1. Furthermore, ERK1/2 promotes the activation of the PI3K/AKT pathway, a major upstream activator of mTORC1 [54,55]. It is already known that HSV-2 inhibits the PI3K/AKT pathway [10]. Our findings demonstrate a decrease in the protein levels of ERK1/2 and mTOR upon HSV-2 infection and IFN-α2B treatment, with further inhibition observed upon co-treatment with HSV-2 and IFN-α2B. Conversely, treatment with a type I interferon inhibitor restored ERK1/2 and mTOR expression, highlighting the role of type I interferon in mTOR inhibition during HSV-2 infection, potentially mediated through the suppression of the ERK1/2 pathway. Mahoney et al. investigated the crosstalk between ERK1/2 and mTOR pathways in non-small cell lung cancer cells with LKB1 and KRAS mutations suggesting that the inhibition of ERK1/2 can lead to the suppression of mTOR signaling [56]. Therefore, the suppression of ERK1/2 by type I interferon signaling, mediated through the induction of suppressor of cytokine signaling (SOCS) proteins, can potentially lead to the downregulation of mTORC1 activity, as ERK1/2 is known to positively regulate mTORC1 through various mechanisms, including the inhibition of the tuberous sclerosis complex (TSC) and the activation of the phosphoinositide 3-kinase (PI3K)/AKT pathway.

It is well-established that multiprotein mTORC1 inhibits autophagy [57,58]. mTORC1 plays a role in modulating autophagy through its influence on the localization and activity of the transcription factor EB (TFEB), a master regulator of genes involved in lysosomal function and autophagy [59]. Studies have demonstrated that mTORC1 directly affects TFEB by phosphorylation at specific sites, Ser142 and Ser211 [59]. These phosphorylation events lead to TFEB being confined to the cytoplasm [60]. Therefore, mTORC1 hinders the overall expression of genes related to lysosomal function and autophagy by phosphorylating TFEB, which results in its cytoplasmic sequestration and consequent inactivation of its transcriptional activity, leading to the inhibition of autophagy. Therefore, our findings suggest that HSV-2 infection induces autophagy through the activation of type I interferon signaling, which inhibits mTOR via the suppression of the ERK1/2 pathway, thereby facilitating viral replication. Thus, HSV-2 modulates the antiviral activity of type I interferon to sustain itself. 

Taken together, our study revealed the downstream mechanisms by which type I interferons induce autophagy upon HSV-2 infection, involving the activation of the JAK-STAT pathway, which inhibits the MAPK pathway, which in turn inhibits mTOR, an inhibitor of autophagy, therefore leading to autophagy upon HSV-2 infection. This study not only highlights the underlying mechanism of autophagy induction during HSV-2 infection but also paves the way for future investigations into potential therapeutic targeting of these pathways to modulate the host–virus interplay. These findings contribute to the understanding of the crosstalk between HSV-2 infection, immune responses, and autophagy, offering insights into the integral molecular dynamics that modulate cellular defense strategies against viral pathogens.

## 5. Conclusions

Our findings demonstrated that type I interferon induces autophagy upon HSV-2 infection, highlighting the mechanisms that dictate the process of autophagy during the HSV-2 viral infection in macrophages and potentially offering therapeutic targets (Figure 9). Autophagy, often considered a cellular defense mechanism, assumes a dual role during HSV-2 infection, contributing to the virus survival and potentially exacerbating the severity of the infection. Our findings demonstrate that the type I interferon pathway serves as a regulator of autophagy during HSV-2 infection in macrophages. Activation of the JAK-STAT signaling pathway by type I interferon leads to the induction of autophagy, providing insights into the unexplored complexity of this process in immune cells upon HSV-2 infection. Furthermore, our study reveals the downstream signaling cascades involved, elucidating how type I interferon finely tunes cellular pathways, including the activation of the JAK-STAT pathway, which leads to the inhibition of the MAPK/ERK1/2 pathway, which in turn suppresses mTOR, a negative regulator of autophagy, ultimately promoting autophagy induction to maintain the survival of HSV-2 virus in macrophages, which has not been demonstrated earlier regarding HSV-2 infection. These findings not only elucidate the underlying mechanisms governing autophagy induction during HSV-2 infection but also pave the way for future investigations into potential therapeutic targeting of these pathways to modulate the host–virus interplay.

## Figures and Tables

**Table 1:** Primer sequences used in the quantitative PCR assays.

Gene	Forward Primer Sequences	Reverse Primer Sequences
UL30	5′-CGCTCAACACGGACTATTACTT-3′	5′-CTCGGTGATCTTGGCGTTATT-3′
IFN-α	5′-AATTCTGCACCGAACTCTACC-3′	5′-ATGGAGTCCGCATTCATCAG-3′
IFN-β	5′-GCTTCTCCACTACAGCTCTTTC-3′	5′-CAGTATTCAAGCCTCCCATTCA-3′
IFN-αβR1	5′-TTAGTGACGCTGTATGTGAGAAA-3′	5′-TGACAAACGGGAGAGCAAATA-3′
IFN-αβR2	5′-GTGAGGAGGGAACACCTTATTT-3′	5′-ATGCACTGAGAAGGCAGATAAA-3′
JAK1	5′-GGATTACAAGGATGACGAAGGA-3′	5′-CGAAGAAGGCCAGGGAAATA-3′
TYK2	5′-CTCAAAGCTGCATCCCTTCT-3′	5′-GTGACTTCCACAAGGACCTAAA-3′
STAT1	5′-CACCTACGAACATGACCCTATC-3′	5′-GCTGTCTTTCCACCACAAAC-3′
STAT2	5′-CAGGCTCATTGTGGTCTCTAAT-3′	5′-GCCCTAGTTCCAGCTCTAATG-3′
IRF9	5′-TCAGAGGTCCCTGGAGTTT-3′	5′-CCCGTTGTAGATGAAGGTGAG-3′
ERK1/2	5′-AGAGAACCCTGAGGGAGATAAA-3′	5′-CGATGGTTGGTGCTCGAATA-3′
BCL2	5′-ATCGCCCTGTGGATGACTGAGT-3′	5′-GCCAGGAGAAATCAAACAGAGGC-3′
BAX	5′-TCAGGATGCGTCCACCAAGAAG-3′	5′-TGTGTCCACGGCGGCAATCATC-3′
GAPDH	5′-CAAGAGCACAAGAGGAAGAGAG-3′	5′-CTACATGGCAACTGTGAGGAG-3′

## Data Availability

All data generated or analyzed during this study are included in this article. Raw datasets [Western blots] are available as the ‘Supplementary Data—Additional file’.

## Supplementary Materials

The following supporting information can be downloaded at: https://www.mdpi.com/article/10.3390/v16091383/s1, Figure S1: Induction of HSV-2 replication upon viral infection; Figure S2: Analysis of cell viability and apoptosis during HSV-2 infection.
