# Supplementary material for: HSV-2 Manipulates Autophagy through Interferon Pathway: A Strategy for Viral Survival
Source: Viruses. 2024 Aug 29;16(9):1383. doi: 10.3390/v16091383 (PMC11437441; doi:10.3390/v16091383)
Supplement: Supplementary file 1 [file viruses-16-01383-s001.zip › viruses-3123135-supplementary.pdf]

Supplementary Figures: viruses-3123135_R1

HSV-2 Manipulates Autophagy Through Interferon Pathway: A Strategy For Viral Survival

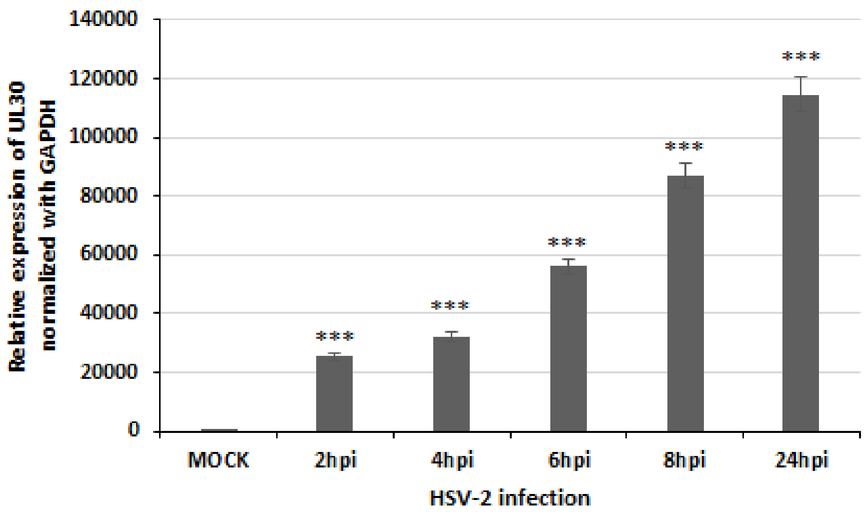

Figure S1. Induction of HSV-2 replication upon viral infection. Relative expression levels of UL30 mRNA, encoding the catalytic subunit of HSV-2 DNA polymerase, in macrophages during HSV-2 infection (MOI 1) compared to mock-infected controls at various time points (2, 4, 6, 8, and 24 hours post-infection, hpi), as determined by quantitative Reverse Transcription-Polymerase Chain Reaction (qRT-PCR) analysis. UL30 expression serves as a marker for active viral replication. Gene expression levels were normalized to GAPDH. Results are presented as the means and standard deviations from three independent experiments. Statistical significance compared to mock: ns, nonsignificant; ***, $p<0.001$.

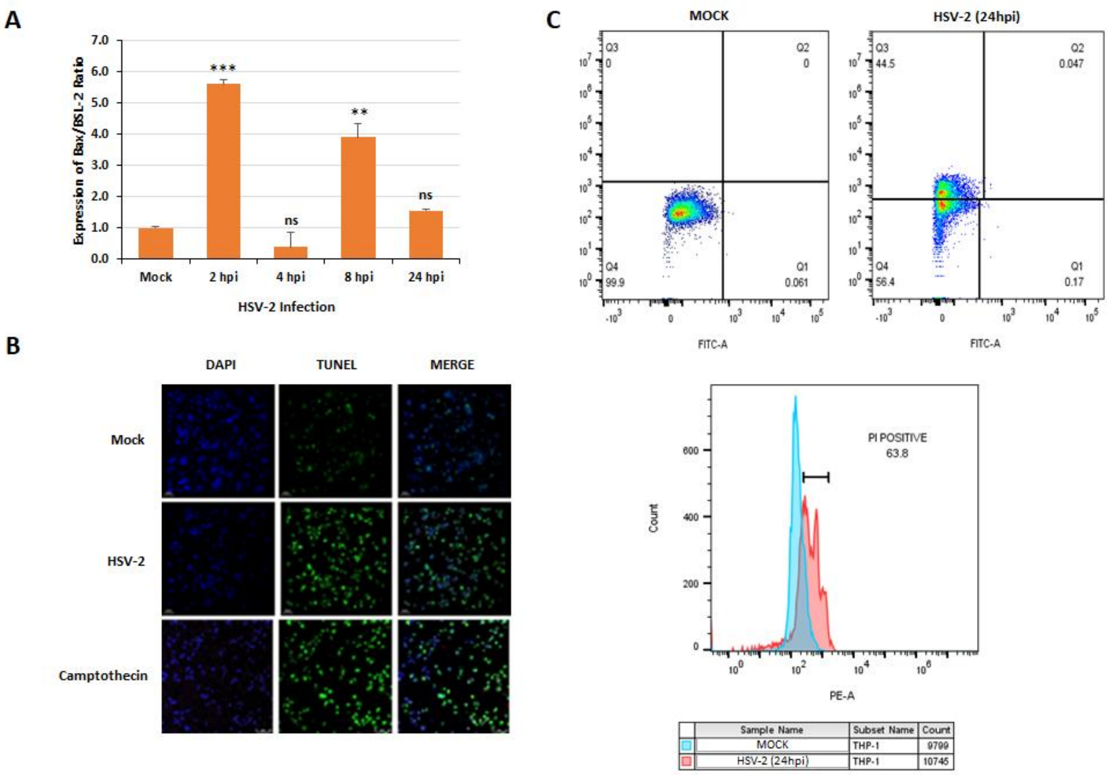

Figure S2. Analysis of cell viability and apoptosis during HSV-2 infection. (A) Relative expression of the cell viability marker gene BCL2/BAX ratio in macrophages during HSV-2 infection (MOI 1) compared to mock-infected controls at various time points (2, 4, 8, and 24 hours post-infection, hpi), as determined by quantitative RT-PCR. Gene expression levels were normalized to GAPDH. Results are presented as means and standard errors from three independent experiments. **(B)** TUNEL assay for detection of apoptotic cells. Fluorescence microscopy images (20x magnification) of mock-infected, HSV-2-infected, and camptothecin-treated (positive control) macrophages at 24 hpi. DAPI (blue) stains nuclei, TUNEL (green) indicates DNA fragmentation in apoptotic cells, and MERGE shows overlaid images. Scale bar: 50 µm. **(C)** Flow cytometry analysis of apoptosis using Annexin V and propidium iodide (PI) staining. Upper panels: Representative dot plots of mock-infected (left) and HSV-2-infected (right) macrophages at 24 hpi. Lower panel: Histogram overlay of PI staining intensity in mock-infected (Blue) and HSV-2-infected (Red) cells. Table shows percentage of cells undergoing Pyroptosis upon HSV-2 infection. Results are representative of three independent experiments. Statistical significance: ns, nonsignificant, ** $p<0.01$, *** $p<0.001$ compared to mock-infected controls.
